# Reliability and validity of the Mexican teachers’ physical activity questionnaire (MTPAQ) in a subsample of female Mexican teachers

**DOI:** 10.1186/s13102-021-00371-4

**Published:** 2021-11-10

**Authors:** C. Medina, A. Monge, M. Romero, R. López-Ridaura, S. Barquera, I. Romieu, E. Denova-Gutiérrez, M. Lajous

**Affiliations:** 1 Center for Nutrition and Health Research, Mexican National Institute of Public Health (INSP), Avenida Universidad 655, Santa María Ahuacatitlán, 62100 Cuernavaca, Morelos, Mexico; 2 Center for Research on Population Health, National Institute of Public Health, Mexico City, Mexico; 3 Center for Research in Evaluation and Surveys, National Institute of Public Health, Mexico City, Mexico; 4 National Center for Preventive Programs and Disease Control. Ministry of Health, Mexico City, Mexico; 5 Section of Nutrition and Metabolism. International Agency for Research on Cancer, Lyon, France

**Keywords:** Reproducibility, Validity, Teachers, Mexico, Questionnaires

## Abstract

**Background:**

Reliable and valid instruments are needed to estimate physical activity levels. The purposes of this study were to estimate the reliability and validity of the Physical Activity Questionnaire (MTPAQ) in a subsample of the Mexican Teachers Cohort study.

**Methods:**

We completed telephone interviews and clinical examinations of 82 teachers. Two MTPAQ, five International Physical Activity Questionnaire (IPAQ)-long form, and two accelerometer (AC) measures were used to determine physical activity levels throughout 24 months. Moderate and walking physical activity (MWPA min/week), vigorous physical activity (VPA min/week), and moderate-to-vigorous physical activity minutes per week (MVPA min/week) were estimated for each instrument. Pearson, Intra-class correlations and deattenuated adjustments were used to determine the reliability and validity of MTPAQ.

**Results:**

MWPA and MVPA min/week of MTPAQs were moderately correlated (r ≥ 0.54) to min/week of IPAQ-long form. MWPA and MVPA min/week average MTPAQ and MTPAQ1 and average AC, AC1 and AC2 were fairly correlated (r ≥ 0.20). A higher correlation was observed when correlation coefficients were attenuated (r ≥ 0.32).

**Conclusions:**

MTPAQ1 is a reliable and valid tool to measure physical activity levels.

**Supplementary Information:**

The online version contains supplementary material available at 10.1186/s13102-021-00371-4.

## Background

Engaging in physical activity is a well-known health behavior that is recommended to prevent and manage certain chronic diseases [1]. In addition, physical activity is a necessary parameter that needs to be estimated in most health studies. The Mexican Teachers Cohort (MTC) is a prospective study of 115,345 female teachers established in 2006–2008 that evaluates lifestyle, including physical activity and environmental risk factors mainly focused on cancer and cardiovascular diseases [2].

Several questionnaires have been developed to estimates physical activity levels [3]. These questionnaires are different with respect to activity domains (leisure, work-related, transportation, household), intensities, energy measure (kcal, METs), time frame (weekend, last week, last month, last year), and style (questionnaire, index, record).[3]However, selection of a questionnaire not only depends on these characteristics but also on the purpose of the study [3].

As part of the MTC, a literature review of instruments that measures physical activity levels was performed. However, none of the available questionnaires were validated in Mexican women nor in teachers, neither had the characteristics and dimension to be used in a cohort study. Thus, the MTC adapted the Stanford Physical Activity Questionnaire [4] to create the Mexican Teachers’ Physical Activity Questionnaire (MTPAQ) [5]. This tool measures the average hours per week spent walking and doing moderate and vigorous (working and leisure time) physical activity in the last 12 months.

Based on previous studies, the mean test–retest reliability for physical activity questionnaires is 0.80 [6]. In addition, a correlation value of 0.30 has been observed between questionnaires and accelerometers [6]. However, this result could be higher if an attenuation adjustment was applied [7]. Until know, there is a lack of information related to psychometric tests of the MTPAQ. The purposes of this study were to determine the reliability and validity of the MTPAQ used in the MTC.

## Materials and methods

### Participants

A random sample of 161 MTC participants aged 35 and older living in Mexico City were invited to participate in the current validation study in 2013. Ninety-nine teachers agreed to participate, signed the consent letter, answered a physical activity, and underwent a clinical evaluation in Mexico City. Written informed consent was obtained from all participants. There were no statistically significant differences in mean BMI, weight, height and waist circumferences, except for age (48 vs. 49 years, *p* < 0.05, respectively) between those that participated and did not in the telephone interviews and clinical examinations.

### Sample size

Based on previous studies, we estimated that at least 30 participants were enough to assess the correlation between IPAQ-long form versus MTPAQ and accelerometer versus MTPAQ [3, 8].

### Procedure

**Fig. 1:**
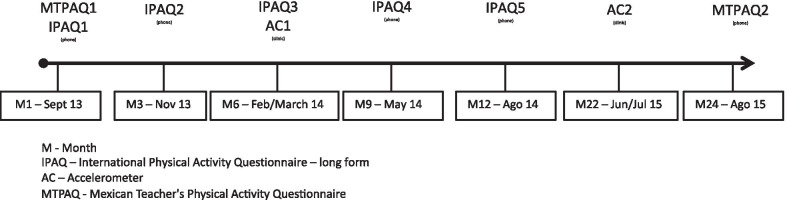
Subjective and objective physical activity assessment through 24 months. *M* month, *IPAQ* International Physical Activity Questionnaire—long form, *AC* Accelerometer

We conducted telephone interviews and clinical examinations of 82 teachers during 24 months. Teachers responded to the Mexican Teacher’s Physical Activity Questionnaire (henceforth: MTPAQ) by phone at month 1 (MTPAQ1) and 24th (MTPAQ2). In the first month, the International Physical Activity Questionnaire (IPAQ)-long form (IPAQ1) was answered by phone and teachers underwent anthropometric tests. A second IPAQ-long form was responded by phone at month 3 (IPAQ2). In the sixth month, teachers received an Actical accelerometer (Mini Mitter Company, Bend, OR, USA). They were asked to wear this device during 9 consecutive days at all the times except when in water activities (ex.: bath, swim). Additionally, a pamphlet that contained instruction on wearing and removing an accelerometer, FAQ, and support contacts were given. Nine days later, accelerometers were removed and another IPAQ-long form was answered (IPAQ3). At months 9 and 12 teachers responded others IPAQ-long form (IPAQ4 and IPAQ5, respectively). Finally, a subsample of 69 teachers used an Actical accelerometer (AC2) for 9 consecutive days during month 22. (Fig. 1). The National Institute of Public Health Ethic Review Board of Mexico approved this study (number 1130).

#### Mexican Teachers Physical Activity Questionnaire (MTPAQ)

**Fig. 2:**
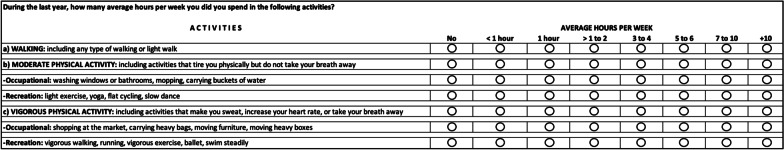
Mexican Teachers Physical Activity Questionnaire (MTPAQ). English version

This is a 5-item questionnaire, and asks for the average number of hours per week spent walking, and doing moderate and vigorous physical activities during work and leisure time over the past 12 months (Fig. 2). The Spanish version is included as a Additional file 1.

Criteria for MTPAQ data cleaning were as follows: (1) data collected in hour per category were converted into minutes: not performing physical activity (0 min), < 1 h (30 min), 1:00 h (60 min), > 1:01 to 2:59 h (120 min), 3:00 to 4:59 h (210 min), 5:00 to 6:59 h (330 min), 7:00 to 10:00 h (510 min), > 10:00 h (600 min); (2) “do not know”, “refused” or “missing data” for duration or frequency were removed from the analysis. As used in the IPAQ, truncation was performed for all daily duration values exceeding 180 min for walking, moderate and vigorous physical activity.

Minutes per week of walking and moderate (MWPA) and vigorous physical activity (VPA) during work and leisure time were summed to create a measure of total moderate-to-vigorous physical activity (MVPA) minutes per week.

#### IPAQ-long form

The International Physical Activity Questionnaire is a 31-item questionnaire that could be self-administered, conducted face-to-face, or answered by telephone [8]. The questionnaire asks about physical activities achieved during leisure time, at home, at yard and at work. In addition, this questionnaire inquiries about active transportation, walking and sedentary activities performed for at least 10 continuous minutes in the previous 7 days [8].

The IPAQ protocol was used to clean up IPAQ values [9]. Information related to data cleaning procedure has been published elsewhere [9].

Minutes per week of moderate (MPA), walking (WPA) and vigorous physical activities (VPA) performed during the four domains (leisure time, home, work and transportation) were summed to generate a measure of MVPA minutes per week.

#### Actical®accelerometer

This is a waistband-mounted omnidirectional device. The Actical accelerometer has been used and validated in adults [10].

The Personal Activity and Location Measurement System (University of California, San Diego, California, United States) and IBM SPSS software; version 24 (SPSS Inc., an IBM company Chicago Illinois, United States) were used to clean the dataset. Compliance criteria was defined based on previous studies [11]. Accelerometer data output is expressed in total counts (counts-min^−1^). These counts were stratified as sedentary (≤ 1.5 Mets, < 100 accelerometer counts), light (1.5 to 2.9 Mets, 100 to < 1500 accelerometer counts), moderate (3.0 to 5.9 Mets, 1500 to < 6500 accelerometer counts), or vigorous (≥ 6 Mets, ≥ 6500 accelerometer counts) [10, 11]. Moderate-to-vigorous physical activities that occurred in ≥ 10 consecutive counts (with allowance of 2 min per each 10 min below the 1530 epoch cut-point) in each valid day were summed to generate VPA, MPA and MVPA minutes per week [11].

#### Physical activity prevalence

This prevalence was estimated for MTPAQs, IPAQs-long form and ACs. WHO physical activity recommendations were used to classified individuals as inactive (< 150 min/week of MVPA) and active (≥ 150 min/week of MVPA) [12].

#### Anthropometry

Weight and height were measured to the nearest 0.1 kg and 0.1 cm, and the Body Mass Index (BMI) was calculated as kg/m^2^. BMI status was based on the WHO adult cut points as: underweight (< 18.5 kg/m^2^), normal weight (18.5–24.9 kg/m^2^), overweight (25.0–29.9 kg/m^2^), or obese (≥ 30.0 kg/m^2^). The BMI variable was divided into two categories: normal weight (< 24.9 kg/m^2^) and overweight/obese (≥ 25.0 kg/m^2^). [13]

### Statistical analysis

Means, standard deviation, interquartile ranges and proportions were used to describe the sample. Physical activity variables were tested for normality using Kolmogorov–Smirnov test and those that did not meet normality criteria were logarithmically transformed. Mean minutes per week between MTPAQ1 and MTPAQ2 were compared using student t-test. Reliability and validity for the log-transformed MPA or MWPA, VPA and MVPA were assessed using Pearson and intra-class correlation coefficients (two-way mixed, average measurements and absolute agreement). Deattenuation adjustment was generated based on the Rosner and Willett recommendations [7]. The 95% CI were obtained by bootstrapping the distribution of means for all PA intensities.

The association between MTPAQ (1, 2, average) minutes per week of MVPA and AC (1, 2, average) minutes per week of MVPA was assessed by a linear regression. The intercept and slopes of the regression line and their associated 95%CI was estimated to observe if values were different from 0 and 1, respectively. Level of significance was set at *p* < 0.05.

Cohen’s kappa coefficients were used to estimate the correlation of the prevalence of physical inactivity/activity between MTPAQ versus IPAQ-long form and MTPAQ versus AC.

For the purpose of this study, coefficient values were classified as follows: < 0.21 were poor, 0.21 to 0.40 were fair, 0.41 to 0.60 were moderate, 0.61 to 0.80 were strong and 0.80 to 1 were very strong [14].

## Results

There were 82 women who answered the MTPAQs, while 86 did the IPAQs, and on average, 61 had two valid accelerometer values. From those that answered both MTPAQs, 42.7% were aged < 45 years, 25.6% had obesity and 36.6% had waist circumference ≥ 90 cm (Table 1).

**Table 1:** Characteristics of participants in a study on the reliability and validity of MTPAQ and IPAQ long-form (n = 82). Mexico, 2013–2015

Variables	N	% (95% CI)
*Age*		
< 45 years	35	42.7 (31.8, 54.1)
≥ 45 years	47	57.3 (45.9, 68.2)
*Body mass index*		
Normal weight	19	23.2 (14.6, 33.8)
Overweight	42	51.2 (39.9, 62.4)
Obese	21	25.6 (16.6, 36.4)
*Waist circumference*		
< 90 cm	52	63.4 (52.0, 73.8)
≥ 90 cm	30	36.6 (26.2, 48.0)

95% *CI* Confidence interval,* N* number

According to Table 2, significant differences were found in VPA minutes per week (164 vs. 101 min, *p* = 0.001). MTPAQ1 and MTPAQ2 minutes per week for all intensities were significantly correlated to each other (*p* < 0.02), ranging from r: 0.26 to r: 0.56.

**Table 2:** Mean minutes per week and standard deviation for the month-specific levels and reliability coefficients for MTPAQ1 and MTPAQ2. Mexico 2013–2015

Variables	1 (n = 82)	2 (n = 82)	Average (n = 82)	(Log min/week) Pearson r (95% CI)
MWPA	376 (300)	332 (268)	354 (237)	0.56* (0.45, 0.88)
VPA	164 (185)*	101 (144)	132 (144)	0.26* (0.04, 0.42)
MVPA	539 (417)	432 (360)	485 (334)	0.54* (0.40, 0.82)

Based on intra-class correlation coefficient (ICC) estimates, a statistically significant correlation was found between MTPAQ1, MTPAQ2 and average IPAQs long-form (range: r = 0.45 to r = 0.80) for all PA intensities. (Table 3) Slightly higher correlations were observed for MWPA and MVPA between average MTPAQs and average IPAQs.

**Table 3:** Pearson, intra-class and deattenuated correlations between log-transformed average minutes per week of log-transformed minutes per week of MTPAQs, five IPAQ long-forms, and two accelerometers. Mexico 2013–2015

	Variables (r (95% CI))		
	Pearson	ICC	Deattenuated
*MTPAQ1 versus Average IPAQ (n* = *86)*			
MWPA	**0.58** **(0.52, 0.99)**	**0.72** **(0.57, 0.82)**	**0.92** **(0.67, 0.99)**
VPA	**0.44** **(0.24, 0.63)**	**0.45** **(−0.09, 0.70)**	**0.99** **(0.89, 0.99)**
MVPA	**0.61** **(0.58, 1.04)**	**0.72** **(0.56, 0.82)**	**0.95** **(0.74, 0.99)**
*MTPAQ2 versus Average IPAQ (n = 75)*			
MWPA	**0.61** **(0.43, 0.79)**	**0.75** **(0.61, 0.84)**	**0.99** **(0.92, 0.99)**
VPA	**0.42** **(0.21, 0.63)**	**0.54** **(0.25, 0.72)**	**0.85** **(0.43, 0.99)**
MVPA	**0.64** **(0.45, 0.81)**	**0.77** **(0.64, 0.86)**	**0.99** **(0.92, 0.99)**
*Average MTPAQ versus Average IPAQ (n = 75)*			
MWPA	**0.64** **(0.51, 0.91)**	**0.78** **(0.65, 0.86)**	**0.99** **(0.99, 0.99)**
VPA	**0.54** **(0.27, 0.57)**	**0.47** **(−0.19, 0.75)**	**0.99** **(0.97, 0.99)**
MVPA	**0.70** **(0.60, 0.97)**	**0.80** **(0.67, 0.88)**	**0.99** **(0.99, 0.99)**
*MTPAQ1 versus Average AC (n = 61)*			
MWPA	**0.25** **(0.17, 0.48)**	**0.37** **(−0.44, 0.62)**	**0.31** **(0.03, 0.57)**
VPA	0.17 (−0.07, 0.37)	0.29 (−0.18, 0.57)	0.29 (−0.09, 0.65)
MVPA	**0.26** **(0.01, 0.36)**	**0.39** **(−0.01, 0.64)**	**0.35** **(0.07, 0.63)**
*MTPAQ2 versus Average AC (n = 59)*			
MWPA	0.18 (−0.08, 0.44)	0.20 (−0.16, 0.48)	0.15 (−0.17, 0.48)
VPA	0.13 (−0.14, 0.39)	0.15 (−0.20, 0.43)	0.16 (−0.24, 0.53)
MVPA	0.21 (−0.05, 0.47)	0.21 (−0.16, 0.49)	0.32 (−0.19, 0.50)
*Average MTPAQ versus Average AC (n = 59)*			
MWPA	0.22 (-0.04, 0.48)	**0.22** **(−0.16, 0.50)**	0.28 (−0.02, 0.55)
VPA	0.22 (−0.04, 0.48)	0.13 (−0.14, 0.39)	0.25 (−0.21, 0.66)
MVPA	0.25 (−0.01, 0.50)	**0.20** **(−0.16, 0.49)**	**0.32** **(0.03, 0.59)**
*MTPAQ1 versus AC1 (n = 87)*			
MWPA	0.16 (−0.06, 0.37)	0.19 (−0.13, 0.43)	**0.22** **(−0.08,0.53)**
VPA	**0.22** **(−0.01, 0.43)**	**0.16** **(−0.14, 0.42)**	**0.39** **(0.02, 0.70)**
MVPA	0.20 (0.01, 0.41)	**0.20** **(−0.13, 0.45)**	**0.30** **(−0.01, 0.56)**
*MTPAQ1 versus AC2 (n = 65)*			
MWPA	**0.33** **(0.09, 0.57)**	**0.33** **(−0.13, 0.61)**	**0.49** **(0.09, 0.86)**
VPA	0.12 (−0.13, 0.37)	0.09 (−0.14, 0.33)	**0.19** **(−0.28, 0.62)**
MVPA	**0.30** **(0.05, 0.53)**	**0.26** **(−0.16, 0.54)**	**0.46** **(0.07, 0.83)**
*MTPAQ2 versus AC2 (n = 63)*			
MWPA	0.14 (−0.12, 0.39)	0.15 (−0.17, 0.42)	0.19 (−0.25, 0.55)
VPA	0.07 (−0.19, 0.33)	0.08 (−0.24, 0.35)	0.12 (−0.39, 0.55)
MVPA	0.15 (−0.11, 0.40)	0.15 (−0.16, 0.41)	0.21 (−0.27, 0.58)

*ICC* intraclass correlation, *MTPAQ* Mexican Teachers Physical Activity Questionnaire, *IPAQ* International Physical Activity Questionnaire—long form, *AC* accelerometer, *MWPA* moderate and walking activities, *VPA* vigorous physical activity, *MVPA* moderate-to-vigorous physical activity, *Bold type* statistically significant correlations

According to Table 3, the correlation between the minutes per week of average MTPAQ, MTPAQ1 and MTPAQ2 and minutes per week of average AC, AC1 and AC2 was statistically significant for MVPA (ranged from r = 0.20 to 0.39) and MWPA (ranged from r = 0.22 to 0.37), except for the correlation between minutes per week of MWPA MTPAQ1 versus AC1 and MTPAQ2 versus AC2. The associations between MVPA and MWPA min/week, in some cases, were higher in the deattenuated correlations.

**Fig. 3:**
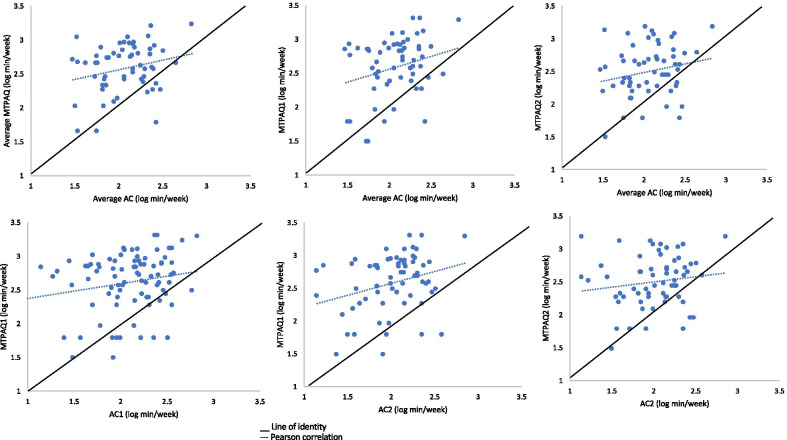
Log transformed minutes per week of MVPA between accelerometers and MTPAQs. Mexico, 2015

As shown in Fig. 3, when MVPA minutes per week of average AC, AC1 and AC2 was used to predict MVPA minutes per week of MTPAQ1, respectively, the intercepts were ranged from 1.41 to 1.62 (*p* < 0.01) and the slopes were ranged from 0.18 to 0.23 (*p* < 0.05). The interception between both lines indicates that teachers within the lowest levels of physical activity observed by average AC and AC2 overestimate self-reported MVPA minutes of MTPAQ1. This overestimation diminishes as accelerometer values increase (interception 692 min and 724 min, respectively). No statistically significant slopes were observed for the MTPAQs versus AC1, MTPAQ1 versus AC1, MTPAQ2 versus ACs and MTPAQ2 versus AC2 (*p* < 0.06).

Cohen’s Kappa coefficient values between ACs and MTPAQs ranged from − 0.02 to 0.12, *p* > 0.05. However, significantly values were observed between MTPAQs and IPAQs-long form ranged from 0.28 to 0.61, *p* < 0.005, being higher the agreement between average IPAQs and average MTPAQs (data not shown).

## Discussion

Our findings indicated that MWPA and MVPA minutes per week of the MTPAQs were moderately correlated (r ≥ 0.54). Moreover, higher correlations were observed in MWPA and MVPA minutes per week between MTPAQ and IPAQ (r ≥ 0.72). Finally, MWPA and MVPA min/week average MTPAQ and MTPAQ1 versus average AC, AC1 and AC2 were fairly correlated (r ≥ 0.20). In some cases, the correlations were higher when the deattenuation adjustment was applied.

Based on previous studies, the reliability for MVPA was lower in our study [15]. Some explanations of this result could be related to the fact that most of the previous studies that reported higher correlation values had a shorter time period between surveys [6, 15]. In addition, a lower correlation was found for VPA compared to other intensities. This contradicts other studies that reported that MWPA showed the lowest reliability estimates [16]. This result could be related to the fact that teachers reported a lower number of minutes of VPA compared to MWPA, and the difficulty in differentiating between intensities.

MVPA and MWPA estimates from the MTPAQ1 and MTPAQ2 were strongly correlated with those reported by the average IPAQ long-form (r = 0.72, 0.77 and r = 0.72, 0.75, respectively). Similar results have been found in a study that measured the correlation between IPAQ long-form and IPAQ short-form in a study of 20 countries (r = 0.67) [8]; a higher correlation was found (r = 0.79) for total physical activity between IPAQ long-form and New Zealand Physical Activity Questionnaire [17]. Some of the explanations of these differences could be the time frame of the recall [18], and the differences in domains between questionnaires [18].

The correlation of the MVPA min/week between the average MTPAQ, MTPAQ1 and average AC, AC1 and AC2 was fair (ranged from 0.20 to 0.39). However, there was no significant correlation of MVPA min/ween between MTPAQ2 and average AC, AC2. Compared to other self-report instruments, diverse correlation values have been observed in different settings and countries for other self-reported physical activity questionnaires (r = 0.14–0.53) [15]. One possibility for the differences in the correlation could be due to the fact that only 2 different weeks may not represent the physical activity levels over 24 months. Other reasons could be participants’ difficulty in estimating their physical activity intensity [19], and social desirability bias, producing an overestimation of physical activity levels [19]. A reason for the non-correlation between MTPAQ2 and average AC, AC2 may be because the MTPAQ2 was answered 1 year and 6 months (AC1) and 1–2 months (AC2) after of the accelerometer measurement. This may not represent physical activity reported by the MTPAQ2.

Some studies have applied an attenuation adjustment to reduce the random error between repeated measurements. These adjustments have been mostly applied to nutrition measurements [7], however, few studies have been published in the physical activity area [20–22]. As shown in this study, other studies that calculated deattenuated correlations in physical activity instruments found that r values seem to be higher compared to correlations that are not deattenuated [23].

### Strengths and limitations

Neither the MTPAQ nor the IPAQ long-form are “gold standard” measures of physical activity, however, the IPAQ long-form could be used to estimate concurrent validity. Based on previous studies, similar estimates have been observed when comparing questionnaires. This is one of the few studies that validated the MTPAQ in a Mexican sample. Although participants used the Actical accelerometer day and night during 7 days, we asked them to remove their device every time they had contact with water. This could result in underestimation of the physical activity. Another limitation of the study includes the long period of time that elapsed between the first and the second MTPAQ and AC measurement. Although we missed physical activity levels measured with the MTPAQ at month 12th, we estimated the correlation between average IPAQ-long form versus MTPAQ1 and MTPAQ2. Finally, this study was limited to Mexican teachers who may not represent all women and teachers from Mexico.

## Conclusion

The MTPAQ had a strong reliability for MWPA and MVPA. High correlation values were observed between MTPAQ and IPAQ-long forms for MWPA and MVPA (r > 60). Minutes per week of MVPA for MTPAQ1 had fair validity values when compared to accelerometer estimates (average, AC1 and AC2). MTC is one of the few women cohort studies in Mexico, this information will serve to estimate and/or correct physical activity levels within the study.

## Supplementary Information

**Additional file 1**. Mexican Teachers Physical Activity Questionnaire (MTPAQ). Spanish version.

## Data Availability

The datasets used generated and analyzed during the current study are not publicly available in any public link but are available from the corresponding author on reasonable request.

## Abbreviations

AC: Accelerometer
BMI: Body mass index
CI: Confidence interval
FAQ: Frequently asked questions
IPAQ: International Physical Activity Questionnaire
Min/week: Minutes per week
MPA: Moderate physical activity
MTC: Mexican Teachers Cohort
MTPAQ: Physical Activity Questionnaire used in the Mexican Teachers Cohort
MVPA: Moderate-to-vigorous physical activity
MWPA: Moderate and walking physical activity
VPA: Vigorous physical activity
