# Supplementary figures and images for: Reliability and validity of the Mexican teachers’ physical activity questionnaire (MTPAQ) in a subsample of female Mexican teachers
Source: BMC Sports Sci Med Rehabil. 2021 Nov 10;13:143. doi: 10.1186/s13102-021-00371-4 (PMC8582186; doi:10.1186/s13102-021-00371-4)

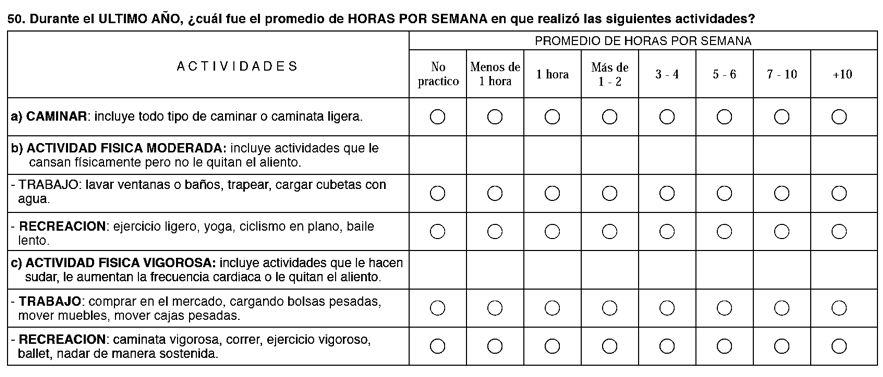

Supplement: Supplementary file 1 — Additional file 1. Mexican Teachers Physical Activity Questionnaire (MTPAQ). Spanish version. [file 13102_2021_371_MOESM1_ESM.png]
